# Improved FTA Methodology and Application to Subsea Pipeline Reliability Design

**DOI:** 10.1371/journal.pone.0093042

**Published:** 2014-03-25

**Authors:** Jing Lin, Yongbo Yuan, Mingyuan Zhang

**Affiliations:** Department of Construction Management, Dalian University of Technology, Dalian, China; Universidad de Valladolid, Spain

## Abstract

An innovative logic tree, Failure Expansion Tree (FET), is proposed in this paper, which improves on traditional Fault Tree Analysis (FTA). It describes a different thinking approach for risk factor identification and reliability risk assessment. By providing a more comprehensive and objective methodology, the rather subjective nature of FTA node discovery is significantly reduced and the resulting mathematical calculations for quantitative analysis are greatly simplified. Applied to the Useful Life phase of a subsea pipeline engineering project, the approach provides a more structured analysis by constructing a tree following the laws of physics and geometry. Resulting improvements are summarized in comparison table form.

## Introduction

Risk assessment is an important aspect of project management in civil engineering and other industrial fields. As its recognition has increased, various methodologies and more specific classifications were developed over the years to assist in risk identification. Reliability risk analysis is considered a part of risk assessment theory.

Reliability risk assessment should be applied throughout the entire lifecycle of a product or structure. Reliability is always the opposite of failure. Risk assessment attempts to quantify probability of failure and in addition the consequences of failure. Therefore analyzing failure modes and mechanisms has become an essential procedure, especially at the beginning of design when corrective actions are most easily incorporated. Failure analysis originated for reactive problem solving or trouble shooting in the manufacturing industry. Internationally, commonly used techniques are Event Tree Analysis (ETA), Fault Tree Analysis (FTA) [1], Failure Modes & Effects Analysis (FMEA), Checklists, What–If Analysis, Preliminary Hazard Analysis (PHA) [2], Cause-Consequences Analysis, Safety Review, Relative Ranking, Human Reliability, Hazard & Operability Analysis (HAZOP), etc. Basically their purpose is to discover and prevent product/structure malfunctions, ensure reliability during the lifecycle and prevent safety hazards while in service.

These approaches follow a standard procedure, starting with the failure description, and then generating hypotheses based on historical data or experience of experts. Hypotheses are grouped into different categories for further calculations and analysis, and finally guide implementation based on qualitative or quantitative conclusions.

In this paper, a new method of reliability risk assessment is developed and applied to a subsea pipeline system. Subsea pipelines are major oil and gas transportation facilities for the deep ocean hydrocarbon mining industry. Pipelines should be designed strong and reliable enough to survive complicated environmental undersea stresses as well as internal and external impacts from both nature and human activities. Impacts could be constant ocean waves, current flow, earthquake or other vibration, corrosion, etc. The consequences of pipeline leakage are destructive and catastrophic for marine life, followed by huge economic loss and environmental cost. For example, in the well-known British Petroleum accident in the Gulf of Mexico in 2010, about 240,000 barrels of crude oil spilled out of three leakage spots along the pipeline per day. 400 different species of life in the area were put at risk. The direct economic loss was over 1 billion USD. In the following year, 2011, an oil leakage disaster caused by a pipeline failure from an offshore oil well shared by ConocoPhillips and China National Offshore Oil Corporation occurred, which contaminated over 170 km^2^ of ocean. It will take decades to clean up the resulting environmental pollution. Clearly it is extremely important to identify reliability failure modes and design them out, if possible, or at least minimize their impact, should they occur. Risk assessment for a subsea pipeline system includes risk factor identification and evaluation, risk control strategies, corrective actions, suitable parameter tolerances and risk monitoring. Such analyses help to build a reliable pipeline system in an effective and economic way.

Domestically in China, a few major methodologies have been applied to risk management of subsea pipeline systems. For example, FTA was developed for subsea pipeline system failure modes by YJ Xie [3]. Analytic Hierarchy Process (AHP) was fitted inside this analysis to evaluate an Expert Scoring Method for reducing data subjectivity. Fuzzy logic analysis has often been used to assess the risk level under various fuzzy conditions [4] and to do criticality assessment of the consequences after a subsea pipeline system fails [5]. [6] used the Fuzzy Relative Matrix method to configure a single factor fuzzy matrix to evaluate the overall safety level of a subsea pipeline system. Based on a commonly used risk assessment system proposed by W. Kent Muhlbauer [7], the root causes of historical subsea pipeline failures fall into four different categories: Third party destruction, Corrosion, Poor design and Operational mistakes. Within each category, further classifications are made for underlying factors. Each factor is rated within its given scope. The sum of the scores is calculated for evaluating the overall risk level of the subsea pipeline system.

A common inadequacy of these methodologies is the lack of sufficient objectivity and their potential for leakage (i.e., missed failure modes). A new method, Failure Expansion Tree (FET), is proposed here for design reference. Significant advantages are shown for its application to subsea pipelines.

## Materials and Methods

### 1 FTA and Traditional Application to Pipeline Reliability Design

The basic FTA method was originated by Bell Laboratories in 1962. It was developed for evaluating the security systems for rocket launching. After that, the airplane maker, Boeing, brought the method to a higher level, both qualitatively and quantitatively [8]. In the following decades, FTA has been recognized and used for reliability analysis and risk evaluation in many industries. For example, FTA has been widely applied in the aviation industry by the U.S. Federal Aviation Administration (FAA) since 1970 [9]. Following the nuclear incident at Three Mile Island, the U.S. Nuclear Regulatory Commission expanded probabilistic risk assessment research, including FTA [10]. In 1992, the United States Department of Labor Occupational Safety and Health Administration (OSHA) published its Process Safety Management (PSM) standard. In 19 CFR 1910.119, FTA was officially accepted as a method for process hazard analysis [11], etc.

**Figure 1:**
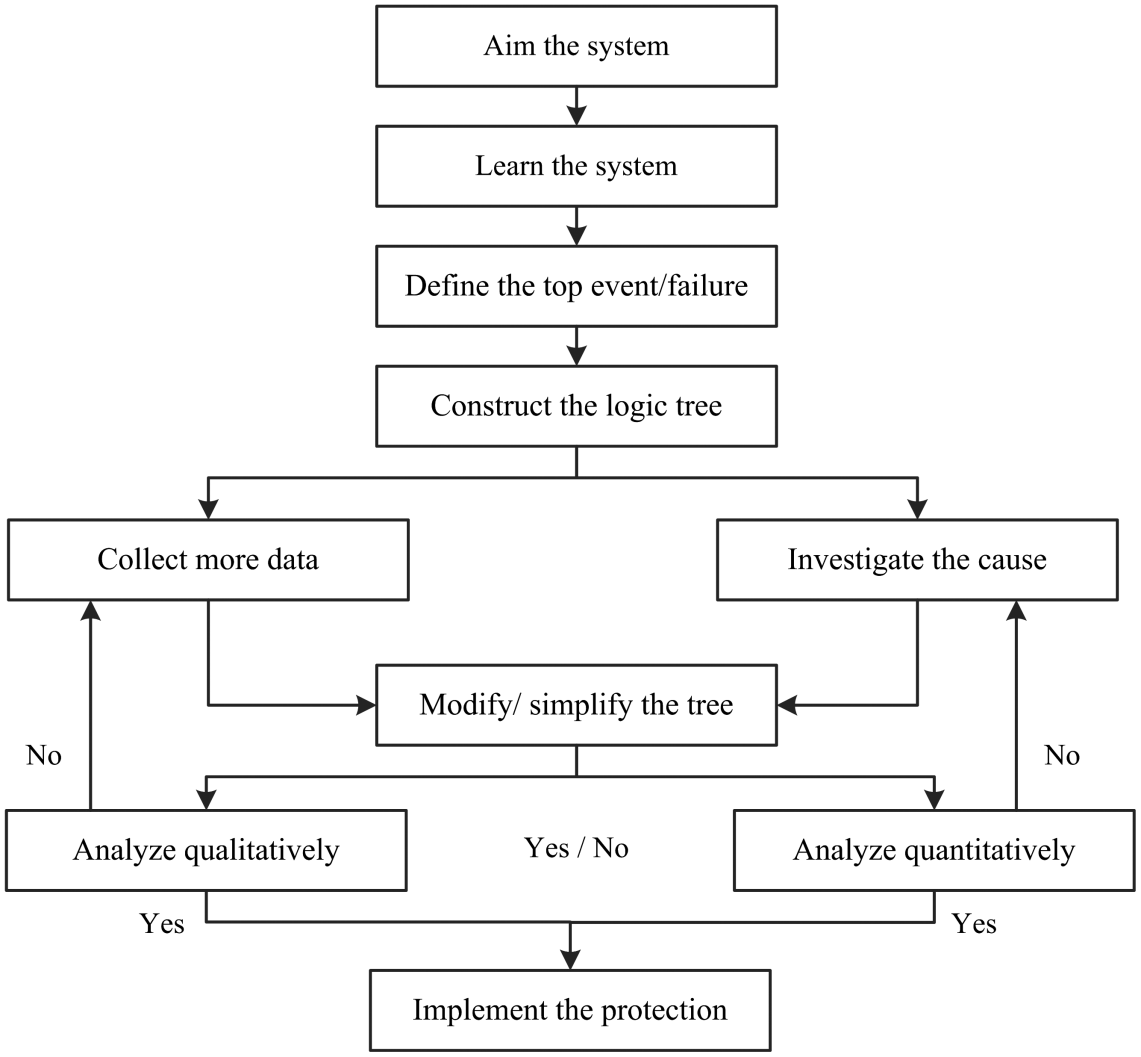
Process Flow Diagram for development of a FTA.

FTA is regarded as an efficient way to describe cause-effect relationships using a logic diagram. It is the starting point for qualitative and quantitative analysis of failure modes. There are five steps to build a FTA: (1) define the problem and classify its boundary, (2) construct the tree, (3) collect the minimum cut/route set, (4) perform qualitative analysis and (5) perform quantitative analysis. The system failure is on the top level of the tree followed by the direct causes (sub-event/sub-factors) on the second level, A1, A2, etc. The two levels are connected by appropriate “And” and “Or” logic gates. The same scheme is used to work further down the tree along each branch until reaching the point that the event/feature cannot be divided anymore. The elements on the very bottom of the tree are called basic elements. Fig. 1 shows a flow chart of the development sequence of a FTA.

Besides [3], FTA examples have been mentioned and demonstrated by various research organizations and utilized in the construction of subsea pipelines. For example, fuzzy fault tree analysis was used for estimating the failure probability of oil and gas transmission pipelines [12] and for evaluating faults from third party damage [13]. In [14] it has been applied to risk assessment for a subsea pipeline under haphazard loads.

**Figure 2:**
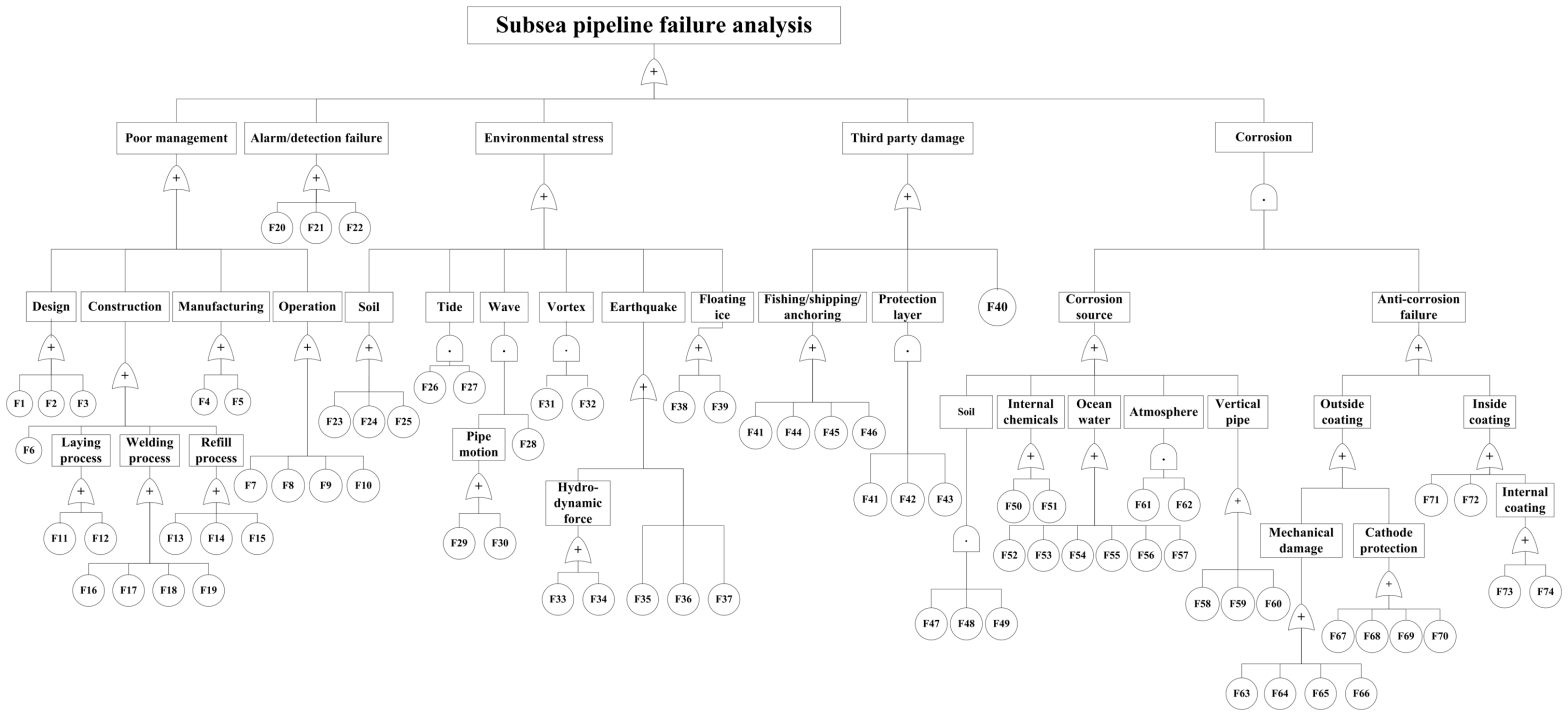
A typical Fault Tree analysis. This example for a Subsea Pipeline System is taken from [3].

The Fault Tree Analysis example in [3] is introduced here to show how the FTA methodology has been applied to subsea pipeline systems. The tree, shown in Fig. 2, codes each failure factor with a number, F(n). A table of these codes is given in Table S2. Note how failure factors are combined with ‘And’ and ‘Or’ gates. Careful inspection of the FTA structure and its codes reveals certain weaknesses which could potentially result in failure mode leakage.

Boxes/factors listed horizontally at a given level occasionally overlap each other, e.g., F23 (low soil viscosity) and F28 (high soil liquidity) are basically stating the same thing, so probability assignments for these “factors” are not independent and cannot be combined with “And” and “Or” gates. Cause and effect are confounded, e.g., F62 the result, “Corrosion in the atmosphere”, and various initiating causes, F63–F66 (anti-corrosion layer damage during construction, shipping, installation and operation) are stated as separate factors. This causes a layer discrimination problem. Another example is the bottom node F67, “Improper cathode protection design”. This statement creates much confusion. Cathode protection damage is NOT simply caused by design, but could be manufacturing, construction, or induced by various environmental stresses. Further inspection of the table reveals other types of logic problems which often result from brainstorming and subjective grouping of possible failure causes.

This example for a Subsea Pipeline System is taken from [3].

### 2 New Logic Tree Proposal

#### 2.1 Tree purposes and objectives

The purpose of traditional FTA is to build a failure analysis model which is useful for both proactive risk control during the design stage and for reactive problem solving/trouble shooting during operating life. A good tree structure should lead to standard conclusions with judgments that are independent of tree builders. In this way, sound design considerations can be generated in a realistic and rigorous manner with minimal leakage during implementation.

#### 2.2 Tree design principles

In order to develop a more complete and less subjective tree, a new methodology, Failure Expansion Tree (FET) analysis is proposed in which certain principles are strictly followed to minimize sensitivity of the bottom line results.

Principle #1: Failure elements (bottom line boxes/nodes) should be events or things physically observable on the structure. The term Failure Mode is defined as “the effect by which a failure is observed on a failed item” [15]. This most obvious and simplest rule has been frequently misused or ignored in daily engineering work. As a result, a misnamed failure could lead the decision maker to incorrect corrective actions and improper sublevel splits. For example, in Fig. 2, in the first level, “poor management” is not a Failure Mode. It is a subjective conclusion drawn by engineer/expert when a failure occurs. The correct description ought to be something directly observed on a physical part. In fact, in this case, “poor management” should not have been listed as a sub-branch. Similar misnaming problems (not all listed) need improvement in this FTA based on Principle #1. By keeping a “common language” between design and problem solving engineers, there would be less confusion and fewer bad assumptions based on differences in understanding.

Principle #2: No bias/prejudgment. Due to prior expert knowledge it is often difficult to draw conclusions without any bias or prejudgment. During failure analysis, instead of beginning with the appearance of the failure directly from the structure and working backwards to its cause, it is easy to leap directly to causes by skipping over the intervening chains-of-events which led to the failure. This ends up mixing failure modes and effects, which then creates difficulty and confusion for further splits in the tree. Taking the example mentioned in Section 2, F65 “Anti-corrosion layer damage”, is the result of “mechanical damage”, instead of the cause of it. It should have been removed from this level. Other “Mechanical damage” nodes (i.e., F63, “Anti-corrosion layer defect caused during construction process, F64, “Anti-corrosion layer defect caused during shipping”, and F66, “Anti-corrosion layer damage caused during operation”) should remain because they are all factors which lead to the mechanical damage. The categories of causes are based on the different physical aspects of the problem (we call these “basis of split”). Again, such modifications can be applied in many other places in Fig. 2 based on this principle.

Principle #3: Boxes must be mutually exclusive. In a logic split, the horizontal elements at the same level should be mutually exclusive to each other, in other words, independent of each other. There should be no overlap of functions or failures between elements within the same level. If such a split cannot be established, it means a higher level split was not properly made. Without this structural requirement interactions between basic elements could be missed later and quantitative calculations based on the tree cannot be correct.

Besides the example mentioned in Section 2 (F23, “low soil viscosity” and F28, “high soil liquidity”), all three splits under the branch of “Design” (“F1, “Poor design model”, F2 “Improper safety parameter values, and F3 “Soil parameters don’t match reality” ) have the same problem. They are confounded with each other, because design model includes safety parameters, and soil parameters. The boxes are not clearly separated. To make the improvement based on Principle #3, “Design” can be split into different aspects of it, such as product development stage, manufacturing stage, or operation stage, which do NOT have overlap functions of each other. And in each stage, different parameters are required to enhance the total reliability of the structure.

Principle #4: Boxes must be collectively exhaustive. Attempting to ensure that all potential failures/risks are covered, the splits on each level should add up to 100% of the possibilities from the immediate node above. This could depend heavily on the way the splits are made. To help make this rigorous, each split must have a “basis” against which to judge this requirement. Typically split bases are physically or logically related to the way the system is organized, not opinions or brainstorm ideas. There are several ways of classifying failure groups by exploiting the objective nature of the system: Function flow, Lifecycle phases, Geometrical features, Time trend, Components, Material composition, etc. Such physical characteristics of a system are independent of the person doing the analysis.

The corresponding improvement in FTA can be demonstrated with the example mentioned in Principle #3 again. The proposed three stages for design cover 100% of the time regime a structure experiences during a full lifecycle. Following this principle, many other splits in Fig. 2 can also be reorganized.

Principle #5: Decompose each node until a physics level is reached where specific corrective actions can be implemented to raise reliability. In the end, only those nodes with the highest relative probability of occurrence require corrective or preventive actions.

In Fig. 2, “Environmental stress” is a very important branch which discovers the external energy threats for subsea pipeline reliability. But the bottom elements reached in this tree didn’t reach the measureable level. To make improvements, for example, F27 “Excess vibration between pipe supports “ can be split further into “direction”, “frequency”, “amplitude”, “time profile” which could be physically measured or tested. This would also provide a parameters list for reliability designers.

Principle #6: Levels developed vertically should be as symmetrical as possible under each parallel node and should follow similar logical sequences and relationships. This provides level independence. If levels are interchanged in a tree, the final quantitative result will be insensitive to level number. This rule should be regarded as a guideline to organize the vertical development of a tree. Layers that are not interchangeable in a tree become sensitive to layer structure by virtue of amplifying the noise associated with probability assignments given to different layer arrangements. Then when the system fails it is commonly attributed to something “unexpected”, or a “rare event” because it was improperly ignored or unforeseen. This principle serves only the quantitative calculation based on the new FET structure. Therefore this is not applicable in the FTA.

The nomenclature, “Failure Expansion Tree”, FET, is chosen to be consistent with Principles#1 and #5 to emphasize the physical and geometrical nature of failures. The word “fault” suggests that human errors are also failure modes. A human’s only role in a “fault” at the design stage is to overlook sources of potential physical failures. Human deficiencies can never be prevented, but physical failures may be prevented, managed or dealt with in some way. However, they must first be recognized. Of course, human error may result in system damage during manufacturing, installation or operation. However, such errors must be dealt with by using preventive methods such as Standard Operating Procedures (SOPs) [16] or Poka Yoke [17], not through fundamental physical design. A failure itself is *always* physical, no matter its originating chain-of-events. Strategies for interrupting a chain-of-events which leads to catastrophic failure, whether human originated or via some minor initiating event, will be the subject of a future paper.

These six principles assure a reliability design with the lowest probability of leakage. It forces people to think how to group things more consistently and logically. Thus, groupings in a FET are physically related and parallel to each other, as opposed to the less-controlled groupings which arise from brain storming and other subjective hypothesis generation methods. Lacking objectivity leads to ambiguous and inconsistent potential corrective actions.

## Results

### 1 Example of FET Application

#### 1.1 Qualitative analysis

**Figure 3:**
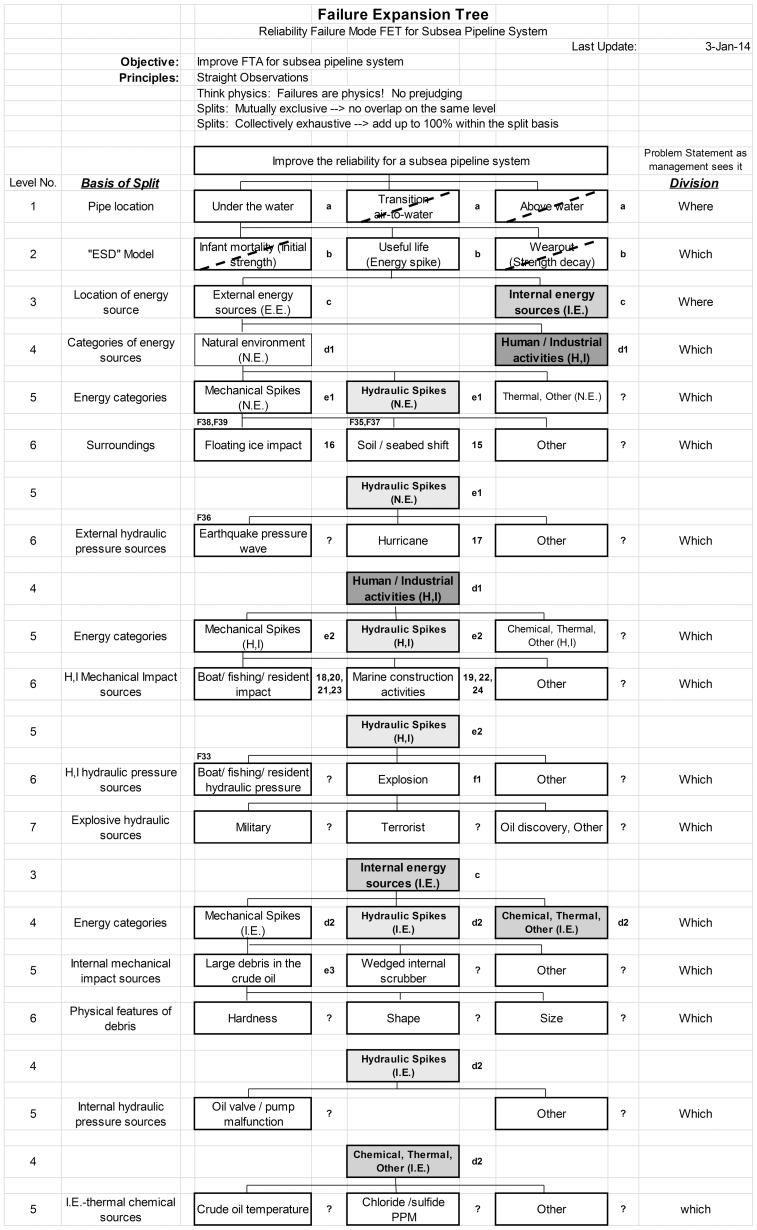
Failure Expansion Tree for identifying the risk factors in a subsea pipeline system during Useful life. Boxes crossed off with a dashed line would be considered in other branches of a complete FET. Failure codes on top of the boxes refer to nodes taken from Reference [3] (see Table S2) and are shown for comparison purposes only. Figures to the right of the boxes refer to probability data taken from [23] and used in the rank order analysis of Fig. 4. Question marks are failure modes unidentified in the original FTA analysis. We call these reliability design “leakage”; they are areas where rare events, being unforeseen, might occur.

We give here an example of a portion of a tree built using the FET principles for one of the three major phases a subsea pipeline structure sees during its service lifecycle. In reliability design, there are three regimes which are generally considered to represent a full lifecycle of a structure: Infant mortality, Useful life and Wear out [18]. In spite of the suggestive names for these three regimes, it is not age that separates them; rather it is the relationship between energies in a system and the strength of a structure to resist those energies. Systems fail when energies exceed strength, whatever the units of energy and strength are and whenever these occur. Infant mortality suggests a weak structure coming from the manufacturing or installation process. Structures fail quickly after being put in the field because normal energies in the environment exceed initial structure strength. Useful life problems result from some kind of energy attack which exceeds the structure’s design strength, resulting in malfunction or destruction. In Wear out, the strength of a structure gradually weakens as it gets older until it can no longer sustain normal environmental energies. It fails naturally towards the end of its life. In the example FET shown in Fig. 3, after making it clear that the focus of this analysis branch is under water, not above or at the water surface, the new tree begins by dividing failures into these three different life cycle regimes.

Fig. 3 shows a partially expanded FET for the “Useful life” regime of a subsea pipeline. We select this regime for an example because it has the longest expected duration in time and is the regime where “rare events” are most likely to occur. An example of an FET for Infant Mortality is given elsewhere [19].

Construction of the tree and its calculation are simply done with a Microsoft Excel Spreadsheet. Down the left hand column of the tree we state the “Basis of Split”, the expectation being that if the basis is clear, then it should be obvious whether one can enumerate all the possibilities defined by it. This is the application of Principle #4, Collectively Exhaustive. Inspection should also make it clear if Principle #3, Mutually Exclusive, has been applied. In the figure we can see how the original FTA nodes (listed above the boxes) have been divided and classified following the mutually exclusive and collectively exhaustive principles, and we can also see where items have been missed.

Examination of the tree reveals that sometimes it is not practical to explicitly list every possibility per Principle #4. For example, within “Energy categories”, which is necessarily an open-ended basis of split, only those types of energies which are reasonably expected to be members of the box immediately above are listed. Other energy types are possible and we remind ourselves of this by including “Other” in the right-most box. However, such abbreviations should be used judiciously because it is important to remember that the primary objective of the FET principles is to force one to think about possibilities that might not otherwise come to mind.

As noted, because the branch expanded in Fig. 3 is Useful life, the focus is on excessive energies, that is, energy spikes which exceed design strength. In a dynamic marine environment, a normally functioning structure might see impacts from numerous such energy sources. In this regime, failure rate is associated with the frequency of occurrence of these sources. In the example we distinguish two geometrically separated energy origins, “External energy sources” and “Internal energy sources”. “External energy sources” are then divided into “Natural environment (N.E.)” and “Human/industrial activities (H,I)”. Under “Natural environment (N.E.)”, the failure code F38 described as “Floating ice compressive strength” from the original FTA belongs to the “Floating ice impact” node in Fig. 3. Since the nature of ice flow is random in location and time (except for seasonality) the structure should ideally survive this kind of event/energy.

While it is clearly not cost-effective to make the pipeline infinitely strong to withstand all ice impacts, recognizing the existence and inability to control such a failure mode may lead one to include an “Inherent Safety” system [20] to limit the effect of any impact to a minor incident rather than allowing a rupture to continue unabated and causing a huge disaster. Such a system could also mitigate the effects of other rare or uncontrollable events, such as earthquakes, tsunamis, terrorism, etc., thus serving “double duty”. Furthermore, such a system should be fail-safe, that is, not requiring a fully operational system in case more than one subsystem is compromised by an energy spike. Multi-subsystem failure was one of the reasons why the March 2011 earthquake in Japan turned the Fukushima Daiichi nuclear power plant failure into such a disaster [21].

We also see missed items in the original analysis. For comparison purposes, missed nodes are denoted with a “?” in Fig. 3. For example “Large debris carried by the crude oil” was not listed. Such “leakage” in the original FTA methodology is one reason why rare/unexpected events creep in. The new FET methodology with its related principles and careful decomposition seeks to solve this problem. Further decomposition of the Debris node provides an example of reaching the decomposition limit of Principle #5. While one cannot control the hardness or shape of unexpected debris in the oil, one could certainly limit its size with some kind of filter and diversion system to remove it, thus improving system reliability and breaking a potentially fatal chain-of-events. With this split under Useful Life we think of debris large enough to cause significant energy spikes from impacts inside the pipe. Following symmetry Principle #6, this same split would appear again under Wear Out where sand or other small particles would cause abrasive wear inside the pipe.

Note that in Fig. 3 all boxes relate to physical things in accordance with Principle #1. Absent are all references to management team quality, regulations, operator skill, poor inspection etc. Such items may be further up the chain of events that allow a failure to eventually develop, however the failure itself is always physical. We must first think of the physical nature of failures. Then either preventive action can be taken during design, or an up-stream system put in place to prevent the failure via some chain-of-events which may or may not involve human error. Again, the FET methodology focuses on the former, which must be recognized first, before the latter can be made effective.

Another significant difference between the FTA and a FET is that the FTA tries to identify not only bottom line elements, but also their interactions. The FET only attempts to find the bottom line individual physical elements. The reason for this is that interactions are often not possible to predict, and it is precisely such interactions that can lead to unexpected results. The philosophy with FET is that unknown interactions among the physical elements must be discovered experimentally through reliability testing using methods such as “multiple environment overstress testing” (MEOST) [22]. Other differences between FTA and FET are summarized in Table S1.

#### 1.2 Quantitative analysis

**Figure 4:**
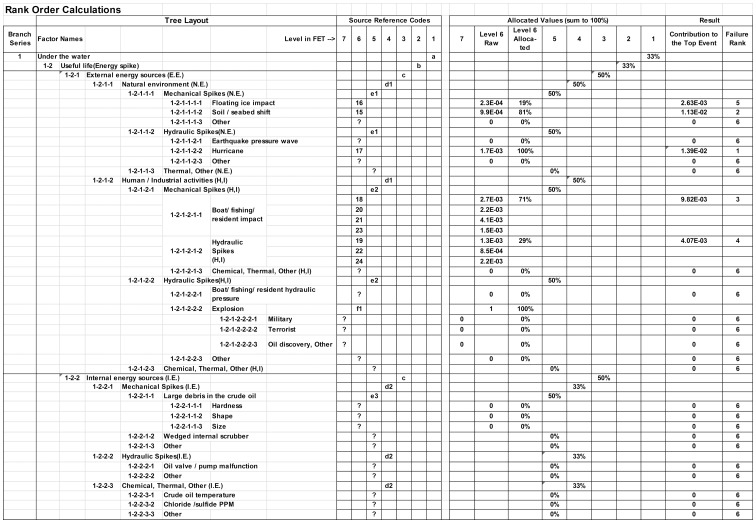
Rank Order Analysis of Useful Life factors. Calculations for ranking are based on the organization of the FET in Fig. 3. Under Source Reference Codes, values are from [23], Table S3. Letters and “?” were not identified or evaluated by the original FTA, so corresponding probability data are unavailable. For demonstration purposes only, missing items are arbitrarily divided equally based on the number of nodes within a given level and“?” are denoted as “0”. Obviously, proper values should be inserted by field experts or suitably researched.

As mentioned, the Useful Life regime was selected here for a calculation example because this is where rare events are most likely to occur. Clearly, there is never enough time or money during the design and installation phases to address all possible failure modes. The purpose of quantitative analysis is, therefore, to rank order the contributions of basic elements to the top event in terms of their relative likelihood of occurrence, and thus provide focus during design for most important reliability issues. Occurrence frequency data can be obtained from prior knowledge and experience in the field or, often helpfully, from other fields which may provide additional insight. Because of the way a failure tree is constructed when following FET Principle #3, there is only one kind of logic relationship involved, the “Or” gate. As shown in Fig. 4, relative probability calculations will therefore contribute multiplicatively through all intermediate levels up to the top level.

Values for the nodes in Fig. 4 are taken either from the FTA example in [23] (Table S3) or they are arbitrarily divided equally within their level for demonstration purposes. Because the methodology requires nodes within a given level to be mutually exclusive and collectively exhaustive, the numerical values are always allocations of 100%, not absolute probabilities. Relative allocation is greatly facilitated by virtue of the fact that each node on a given level has the same “basis”, so the elements are directly comparable to each other. After multiplicatively calculating top-level contributions, the values are rank ordered on the far right. Fig. 4 is an image cut directly from an Excel spreadsheet.

In practice, a Failure Expansion Tree (like a FTA) will expand very quickly as the decomposition progresses. During decomposition, probabilities would be updated for all the current bottom nodes and then further decomposition would focus only on those which are among the top, say, ∼25% of all current bottom nodes. Rank order of the top dozen nodes or so would be kept. When those dozen are all down to controllable physical factors, then the decomposition is complete and corrective actions can be taken top-down on the rank ordered list using whatever time and resources are available.

A Normal (i.e., Gaussian) distribution is often assumed in failure probability distribution studies. However, a problem arises with this assumption. It grossly underestimates the probability of rare events whose tails follow a power law distribution [24]. The long power law tail results from the accumulation of all ignored, missed or improperly evaluated factors. The improved FET is more likely to list all the factors because of the way it is constructed and the ranking is more realistic because of the way probabilities are allocated. Thus, the close-in parts of the power law tail are more likely to be included in the top dozen ranking, and therefore dealt with during design. This results in improved reliability in the face of what would otherwise be considered a rare event.

Notice that quantitative analysis of the FET only covers the probability of occurrence of the top level event. In a large and complex system, multiple subprojects or subsystems should be identified and treated individually to make the analysis manageable. The objective of each subproject is to reduce occurrence of its own top level event.

Finally, for proper risk assessment, the ultimate decision of what should be improved in the design should be made based on the product of the calculated “Contribution to the top event” and its associated potential economic loss. Economic loss evaluation requires another round of analysis, not shown in Fig. 4, nor discussed in this paper.

Some additional comments about Fig. 4 are worth noting:

1. According to the rank order calculation, the top 5 factors requiring the most attention are: “Hurricane”, “Soil/seabed shift”,“Boat/fishing resident impact”, “Hydraulic Spikes(H,I)”, and “Floating ice impact”. All of these areas can (and probably should) be divided further into measurable and controllable levels. For example, “Boat/fishing resident impact” could be divided in ways which might suggest specific methods to measure, detect and prevent occurrence. If failure is considered unavoidable, automatic rupture detection and shutdown systems should be incorporated to mitigate a potential disaster.
2. The original FTA analysis in [23] concluded the top 5 factors were “Third party damage”, “Corrosion”, “Vortex-induced vibration”, “Management”, “Operation”. While corrosion would belong to the “Wear Out” branch of a complete FET, the ranking is very different between the FET and the FTA. This is partly due to the different way the FET decomposition proceeds with its focus on the physics of failures, but in this example it is also largely due to the limited availability of data from prior work for the FET calculation. Where a “?” was encountered, the item is missing in the original FTA’s and no attempt was made to quantify it. We simply denoted it as “0”. Clearly, allocations must be developed for each FET level and node based on the frequency of occurrence of comparable nodes or other relative estimations.
3. The Useful life branch of a complete FET is relatively short compared to branches of the other two regimes, mostly because there are fewer spike energy sources than there are ways things can go wrong during manufacturing, installation or Wear out. For example other energy sources such as biological, chemical/corrosive or abrasive would all fall under Wear out because they accumulate over time, but would not produce energy spikes. Initial strength weaknesses such as porous welds, dents, or low metallurgical hardness would fall under Infant mortality. Strength is not considered at all under Useful life because in this regime we only look for energies that exceed the design strength, not the actual strength. If normal energies exceed actual strength of some parameter which was not considered in the design, this would fall under Infant Mortality. Improved reliability would result from identifying this parameter, hopefully first with an Infant Mortality FET, and then strengthening it. However, multi-layer protective analysis would also call for use of techniques such as MEOST to identify unforeseen weaknesses should they be missed up front, even with a FET analysis.

#### 1.3 Sensitivity of quantitative calculations

**Figure 5:**
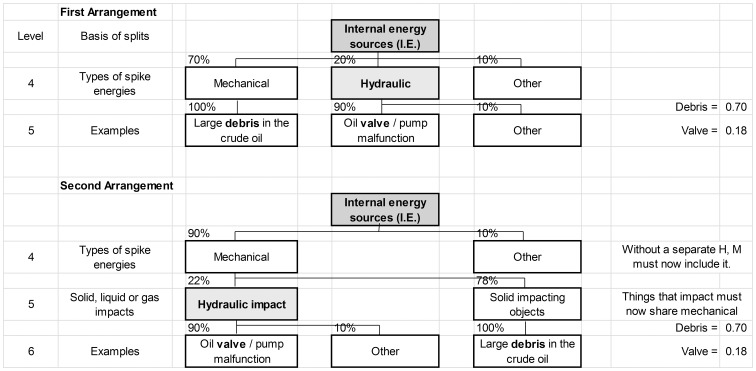
Demonstration of calculated results from two different tree structures with interchanged layers. On top of each box, the number is the proposed percent allocation with respect to the box above it. Node probability is the product of all layers above it and is calculated on the right hand side for Debris impact and Valve failures.

Based on Principle #6, layers in the FET should be reasonably interchangeable without significantly affecting the quantitative calculation results, and therefore ranking. In other words, results should be relatively insensitive to tree layout as long as that layout follows a physical and logical sequence. The example in Fig. 5 demonstrates how this is achieved with a FET.

In the first arrangement “Hydraulic” is listed on level 4, parallel to “Mechanical” and “Other”. It is rated as 20% of the total with respect to “Internal energy sources”. In terms of frequency of occurrence, one might think of this data coming from experts having seen 7 mechanical events, 2 hydraulic events and 1 other kind of event, giving rise to the 70-20-10 percent split. Similarly, “experience” would have given rise to the other values shown. Under this tree arrangement, the “Debris” ranking score will be 100% × 70% = 0.7, while the “Valve” ranking score will be 90% × 20% = 0.18.

In the second arrangement, “Hydraulic” is considered a part of “Mechanical”. It is now thought of as only one type of mechanical force (a conforming one) and would be listed in parallel with, say, “Solid impacting objects”. This arrangement adds an intermediate layer between levels 4 and 6 with the split basis, “Solid, liquid or gas impacts” (the relevant possible states of impacting matter). “Mechanical” at level 4 must now be 90% since it subsumed “Hydraulic”, while “Other” remains at 10% (or 9 events vs. 1 event in terms of frequency). “Hydraulic” itself was originally 2 events, while large debris was 7 events (100% of Mechanical in the first arrangement). These two add up to a total of 9 events. Normalizing the values to 100% gives us an allocation of 22% for Hydraulic and 78% for Solid Objects. The final calculation in this arrangement is “Debris” = 90% × 78% × 100% = 0.70 and “Valve” = 90% × 22% × 90% = 0.18, exactly the same as in the first arrangement.

There are two reasons that interchanging levels does not significantly affect the final ranking of each node. First, all the nodes in a given level must to add up to 100% through allocation. Second, where node data is available as a frequency of occurrence at the top level, that node’s relative ranking with respect to other comparable nodes at the same level is always fixed, regardless of the level at which it appears in the FET.

Notably, the allocation approach within levels makes it easier to judge relative contributions for boxes for which factual data may not be readily available. Comparison of equivalent things is always easier than making absolute statements.

## Discussion

Table S1 compares nine different aspects of the two methodologies. The advantages and disadvantages of each methodology are shown in column 2 and 3. The 4th column lists which principles have been applied to make the improvements, and explains how they work.

## Conclusions

A new technique for improving Fault Tree Analysis, FTA, has been presented and applied to improving submarine pipeline reliability during its useful life. The original FTA nodes from [3] were filtered, reconstructed, revised and extended by following a logical sequence of physical decomposition using six key principles aimed at reducing failure mode leakage, instead of brainstorming or use of other subjective risk factor identification methods. The improved tree is called a Failure Expansion Tree, or “FET”, suggesting a focus on physics and geometry. The calculation of each risk factor’s relative contribution to the top event is carried out with a Microsoft Excel Spreadsheet. No “least cut set” is needed for massive calculation as in FTA. Simple “Or” logic is used throughout the new tree, which largely reduces the complication of computer programming and clarifies failure routes. Besides helping one focus during the design phase, the structure also enhances the decision maker’s ability to quickly review and react later during problem solving.

The six principles for building a Failure Expansion Tree are: (1) failures are things that are physically observable on parts, (2) avoid confounding cause and effect which often results from skipping over an intervening chain of events, (3) split boxes must be mutually exclusive to avoid overlap, (4) they must be collectively exhaustive and decomposed according to the physics and geometry of the structure to assure completeness, (5) nodes must be decomposed until corrective action can be taken at a physical level and (6) trees must have an element of symmetry to avoid quantitative sensitivity to level structure. A key advantage of the FET approach is to achieve more complete risk factor coverage, and thus uncover potential rare events. Rank order calculations allow focusing improvement work on the “Top 5” or so factors which have the highest relative probability of occurrence, or the highest cost if failure occurs. In the example presented, the FET approach was more likely to identify events caused by energy spikes during useful life. Addressing these will achieve improved reliability.

The example in this paper was for methodology illustration purposes only. A practical application with more comprehensive splits down to addressable physics levels and proper quantitative data would require suitable industry experts.

## Supporting Information

Table S1 FTA vs. FET.
(DOCX)

Table S2 Description of various reference codes from a subsea pipeline fault tree [3] (probability data not available).
(DOCX)

Table S3 Description of various fault codes with probability values from a subsea pipeline fault tree [23].
(DOCX)
