# Supplementary material for: Improved FTA Methodology and Application to Subsea Pipeline Reliability Design
Source: PLoS One. 2014 Mar 25;9(3):e93042. doi: 10.1371/journal.pone.0093042 (PMC3965535; doi:10.1371/journal.pone.0093042)
Supplement: Table S1 — FTA vs. FET. (DOCX) [file pone.0093042.s001.docx]

Table S1. FTA vs. FET

|  | FTA | FET | Corresponding improvement mechanism |
| --- | --- | --- | --- |
| 1 | Splits are NOT “collectively exhaustive”. There is no criterion to assure completeness in any sense. | Splits are “collectively exhaustive” which help assure completeness of the analysis and identification of potential rare events. | Principle # 4 is applied to improve FTA. Factors in the same level add up to 100%, |
| 2 | People with different expertise often come up with different FTAs based on subjective opinions. | Tree follows a logically consistent process based on system physics and geometry, which are necessarily invariant and not subjective. | Principle #1 is applied. Physics observation and logic don’t change because of different personnel. |
| 3 | Complicated weighting of individual factors confounded by incompleteness and overlap. | Decomposes industrial experience downward through allocation, not rolling guessed probabilities upwards. | Principle #3 keeps factors separated from each other without overlapping them. Principle #2 is used to avoid subjective grouping among layers. |
| 4 | Bottom up thinking: think of causes (X), then group them. Allows confounding elements in the chain-of-events. | Top down decomposition thinking - effect to cause. Focuses only on the physics and geometry of failures. Avoids confounding. | Principles #2&5 are applied to clear up layer discrimination and decompose each element down to a measurable level-X (cause) |
| 5 | Node identification is based on haphazard brainstorming and subjective opinions. | FET decomposition proceeds systematically. Rare events are less likely to be overlooked. | Principles #1-4 are applied to construct a tree more cleanly, completely and systematically. |
| 6 | Factors on each level have no basis of comparison (they are not mutually exclusive). Decisions are based on guessed probabilities. | Factors on each level are not mixed (no apples vs. oranges). They must share the probability from the node immediately above. | Basis of split is created for each level to guarantee principle #3&4 are implemented. |
| 7 | System becomes more complex if additional subsystems are added, which potentially leads to LOWER, not higher reliability. | No difference with FTA. However FET is a better way of sorting the “top 5”. At least design complexity increases preferentially for the most likely/costly causes. | Both trees are expansion type. FET expands systematically by following 6 principles. |
| 8 | Intends to discover both individual factors and their interactions. However, factors are easily confounded, and interactions cannot be easily predicted. | Focuses only on discovering individual factors through logic and physics. These factors are fed forward into reliability tests to discover interactions experimentally. | All 6 principles help improve coverage for failure factors. Finding interactions requires a different approach. See 4.1 |
| 9 | Relies heavily on mathematics and computer programs to deal with interactions resulting from the need of “And” and “Or” gates. | Only requires use of “Or” gates so probability and ranking calculations are greatly simplified. “And” only makes sense when interactions are analyzed after FET is completed. | Principle #3 provides clear factor separation so the only relationship between them is “or”. |
