# Supplementary material for: Improved FTA Methodology and Application to Subsea Pipeline Reliability Design
Source: PLoS One. 2014 Mar 25;9(3):e93042. doi: 10.1371/journal.pone.0093042 (PMC3965535; doi:10.1371/journal.pone.0093042)
Supplement: Table S2 — Description of various reference codes from a subsea pipeline fault tree [3] (probability data not available). (DOCX) [file pone.0093042.s002.docx]

Table S2. Description of various reference codes from a subsea pipeline fault tree [3] (probability data not available)

| F01 | Poor design model | F38 | Floating ice- compressive strength |
| --- | --- | --- | --- |
| F02 | Improper safety parameter values | F39 | Floating ice too thick |
| F03 | Soil parameters don’t match reality | F40 | Inadequate pipe inspection |
| F04 | Poor incoming material quality | F41 | Water too shallow |
| F05 | Poor incoming inspection | F42 | Poor quality of pipe coating layer |
| F06 | Damage during pipe shipping | F43 | Improper pipe coating structure |
| F07 | Poor management team quality | F44 | Soil on top of pipe too thin |
| F08 | Poor management team attitude | F45 | Busy ocean transportation activities |
| F09 | Unstable management team | F46 | Development of fishing industry |
| F10 | Improper management regulations | F47 | Low soil electrical resistance |
| F11 | Pipe damage during horizontal laying | F48 | Electrical charges |
| F12 | Pipe damage during vertical laying | F49 | Bacterial corrosion |
| F13 | Improper soil filling process | F50 | High O2 content of water in the oil |
| F14 | Insufficient soil filling | F51 | Sand an organic compounds in the oil |
| F15 | Wrong soil filling material | F52 | High O2 in the ocean |
| F16 | Unprofessional welder | F53 | High ocean microbe activity |
| F17 | Improper welding process | F54 | High salt concentration in the ocean |
| F18 | Poor welding inspection equipment | F55 | High ocean water flow rate |
| F19 | Poor welding inspection skill | F56 | Low ocean water temperature |
| F20 | Inspection gage failure | F57 | Low ocean water pH |
| F20 | Unstable inspection gage | F58 | Corrosion of the vertical part of the pipe (above the soil) |
| F22 | False/invalid alarm | F59 | Corrosion of the vertical part of the pipe(in the soil) |
| F23 | Low soil viscosity | F60 | Corrosion of the vertical part of the pipe (above the water) |
| F24 | Poor soil anti-shearing strength | F61 | Atmospheric induced corrosion |
| F25 | Soil on top of pipe too thick | F62 | Corrosion in the atmosphere |
| F26 | Shifting sea bed soil | F63 | Anti-corrosion layer defect caused during construction process |
| F27 | Excess vibration between pipe supports | F64 | Anti-corrosion layer defect caused during shipping |
| F28 | High soil liquidity | F65 | Anti-corrosion layer defect caused during installation |
| F29 | Soil filling too shallow | F66 | Anti-corrosion layer damage caused during operation |
| F30 | Pipe material density too low | F67 | Improper cathode protection design |
| F31 | Reynolds number too big | F68 | Improper cathode inspection |
| F32 | S value too high | F69 | Poor cathode maintenance |
| F33 | Drag force created by ships | F70 | Improper cathode operation |
| F34 | Inertial force created by ships | F71 | No or misused inhibitors |
| F35 | Earthquake axial direction forces | F72 | No protection of pipe inner layer |
| F36 | Earthquake inertial forces | F73 | Internal coating voids |
| F37 | Earthquake pressure from the soil | F74 | Disbonding of internal coating |
