# Supplementary material for: Improved FTA Methodology and Application to Subsea Pipeline Reliability Design
Source: PLoS One. 2014 Mar 25;9(3):e93042. doi: 10.1371/journal.pone.0093042 (PMC3965535; doi:10.1371/journal.pone.0093042)
Supplement: Table S3 — Description of various fault codes with probability values from a subsea pipeline fault tree [23]. (DOCX) [file pone.0093042.s003.docx]

Table S3. Description of various fault codes with probability values from a subsea pipeline fault tree [23].

| 1 | Poor pipelines safety | 8.63x10^-3^ | 39 | Improper anti-corrosion layer design | 1.13x10^-3^ |
| --- | --- | --- | --- | --- | --- |
| 2 | Poor construction system safety | 2.32x10^-4^ | 40 | Poor anticorrosion layer quality | 5.23x10^-4^ |
| 3 | No fatigue prevention | 2.89x10^-4^ | 41 | Poor anticorrosion layer inspection | 1.13x10^-3^ |
| 4 | No allowance for water impact | 7.28x10^-4^ | 42 | Poor anticorrosion layer maintenance | 1.71x10^-3^ |
| 5 | Poor testing of hydraulic pressure | 8.63x10^-3^ | 43 | Highly corrosive compounds in the crude oil | 8.63x10^-4^ |
| 6 | Pipe movement through shifting soil | 2.41x10^-3^ | 44 | Internal anticorrosion layer damage | 2.41x10^-3^ |
| 7 | Pipe movement through flushed soil | 5.00x10^-3^ | 45 | No cathode protection | 1.69x10^-3^ |
| 8 | Pipe movement through regions of high dynamic energy | 8.43x10^-4^ | 46 | Poor cathode protection | 1.50x10^-3^ |
| 9 | Thin seabed layer | 5.16x10^-4^ | 47 | Cathode system age | 1.91x10^-3^ |
| 10 | High soil penetration | 1.15x10^-1^ | 48 | Highly corrosive sea water | 2.70x10^-3^ |
| 11 | High soil saturation | 9.92x10^-4^ | 49 | Interaction with nearby metals | 9.92x10^-4^ |
| 12 | Tide impact | 8.53x10^-4^ | 50 | Mechanical degradation | 8.53x10^-4^ |
| 13 | Ocean wave impact | 5.16x10^-4^ | 51 | Pipeline material aging | 2.70x10^-3^ |
| 14 | Vortex impact | 1.91x10^-3^ | 52 | Insufficient electric potential | 1.71x10^-3^ |
| 15 | Earthquake impact | 9.92x10^-4^ | 53 | Intermittent measurements | 6.04x10^-3^ |
| 16 | Floating ice impact | 2.32x10^-4^ | 54 | No internal inspection or poor measurement equipment | 6.23x10^-4^ |
| 17 | Hurricane impact | 1.69x10^-3^ | 55 | Poor risk identification during design | 7.26x10^-3^ |
| 18 | Frequent shipping activities | 2.70x10^-3^ | 56 | MAOP reached during normal operation | 2.15x10^-3^ |
| 19 | Dredging activities | 1.32x10^-3^ | 57 | No safety design | 1.15x10^-3^ |
| 20 | Anchoring activities | 2.15x10^-3^ | 58 | Poor choice of pipe material | 8.53x10^-4^ |
| 21 | Fishing activities | 4.11x10^-3^ | 59 | Design not proven by experts | 3.71x10^-3^ |
| 22 | Construction near the pipeline | 8.53x10^-4^ | 60 | Poor inspection during pipe installation | 6.23x10^-4^ |
| 23 | Residential activities | 1.48x10^-3^ | 61 | Uncertainty of pipe material utilization | 1.48x10^-3^ |
| 24 | Interference with nearby construction | 2.15x10^-3^ | 62 | Poor welding inspection | 8.53x10^-4^ |
| 25 | Shallow water | 6.16x10^-4^ | 63 | Improper soil filling | 5.47x10^-3^ |
| 26 | Shallow burial of the pipeline | 2.41x10^-3^ | 64 | Improper shipping/handling | 3.55x10^-4^ |
| 27 | Concrete layer damage | 7.95x10^-3^ | 65 | Poor protective layer installation | 4.11x10^-3^ |
| 28 | Ship guiding path broken | 1.15x10^-3^ | 66 | Lack of strict operating regulations for key equipment | 7.28x10^-4^ |
| 29 | Protection wrap damage | 1.71x10^-3^ | 67 | No SCADA communication system | 5.50x10^-3^ |
| 30 | Poor public awareness | 7.28x10^-4^ | 68 | No employee safety for toxic and hazard situations | 8.53x10^-4^ |
| 31 | No/poor direct alarm system | 4.44x10^-4^ | 69 | Poor safety plan | 1.50x10^-3^ |
| 32 | No/poor pipeline signage | 1.30x10^-3^ | 70 | Poor inspection during operation | 5.23x10^-4^ |
| 33 | Poor pipeline inspection | 6.23x10^-4^ | 71 | Poor employee training | 2.70x10^-3^ |
| 34 | Poor pipeline inspector skill | 3.35x10^-3^ | 72 | No mechanical error protection | 4.54x10^-3^ |
| 35 | No/malfunctioning sensing equipment | 8.53x10^-4^ | 73 | Poor maintenance documentation | 2.15x10^-3^ |
| 36 | No or infrequent inspection | 3.01x10^-3^ | 74 | Poor maintenance plan | 6.04x10^-3^ |
| 37 | Exposure to a corrosive environment | 9.80x10^-4^ | 75 | No documented maintenance procedures | 6.23x10^-4^ |
| 38 | Highly corrosive atmosphere | 8.63x10^-4^ |  |  |  |
